# Fatigue endurance enhancement of Sn-doped Pb(Lu_1/2_Nb_1/2_)O_3_–PbTiO_3_ ceramics

**DOI:** 10.1039/c8ra00732b

**Published:** 2018-03-26

**Authors:** Chenxi Wang, Chao He, Zujian Wang, Xiuzhi Li, Xiaoming Yang, Ying Liu, Xifa Long

**Affiliations:** Key Laboratory of Optoelectronic Materials Chemistry and Physics, Fujian Institute of Research on the Structure of Matter, Chinese Academy of Sciences Fuzhou 350002 Fujian China hechao@fjirsm.ac.cn lxf@fjirsm.ac.cn; University of Chinese Academy of Sciences Beijing China

## Abstract

Several mechanisms and methods have been proposed to study the nature of electric fatigue in ferroelectric materials with perovskite structure, including defect agglomeration, field screening and the reorientation of defect dipoles. To ascertain the effect of defect, defect dipoles in particular on the fatigue behavior in perovskite ferroelectrics, 0.51Pb(Lu_1/2_Nb_1/2_)O_3_–0.49PbTi_1−*x*_Sn_*x*_O_3_ ferroelectric ceramics were fabricated in this work. It is found that the fatigue endurance has been enhanced after Sn-doping. An abnormal strong self-rejuvenation of polarization was also detected for un-poled and un-aged samples resulting from the reorientation of defect dipoles. The defect dipoles were determined by the confirmed change of the valence of Sn ions and the appearance of oxygen vacancies. The reorientation was also confirmed by the internal bias of *P*–*E* hysteresis loops during the fatigue process. With more Sn doped into the matrix, the symmetry changed from a coexistence of rhombohedral and tetragonal phase to a rhombohedral phase. The remnant polarization decreased, while the coercive field first decreased then increased as *x* increased, which resulted from the composition variance and the effect of defect dipoles. It indicates that the defect dipoles play an important role in the electric fatigue behavior of Sn-doping PLN–PT ceramics.

## Abstract 2

Fatigue endurance has been enhanced of Sn-doped Pb(Lu_1/2_Nb_1/2_)O_3_–PbTiO_3_ ceramics, exhibiting an abnormal self-rejuvenation of remnant polarization.

## Graphical abstract

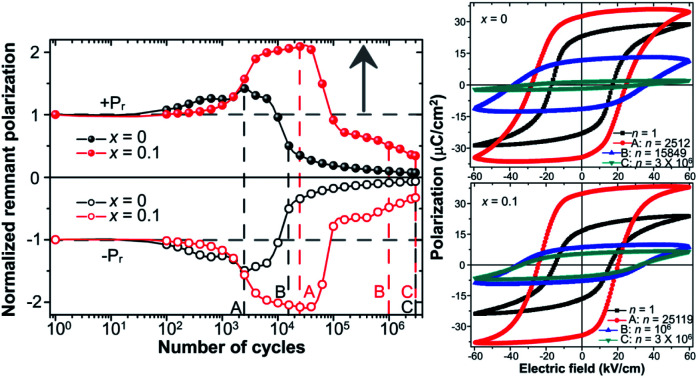

## 1. Introduction

Ferroelectrics with perovskite structure have been the cornerstone of modern high-tech electrical devices such as actuators, piezoelectric transducers and acoustic transducers.^1–3^ Among various ferroelectrics with a complex perovskite structure, Pb(Lu_1/2_Nb_1/2_)O_3_–PbTiO_3_ (PLN–PT) solid solutions were found in which the morphotropic phase boundary (MPB) between tetragonal and rhombohedral phase is in the range of 0.49–0.51PT, exhibiting excellent electrical properties and high Curie temperature (*T*_C_),^4–6^ which makes the PLN–PT system a promising candidate for high-power transducer applications with a wide range of application temperature. In actual applications, devices are subjected to repetitive electric cycles. The severe degradation of reversible polarization and subsequent degradation of various electric properties will be caused by cyclic electric field, *i.e.* electric fatigue. Electric fatigue hinders the reliability and life length of ferroelectric devices severely.^7^ Hence, a good electric fatigue endurance is highly desired for ferroelectric devices.^8^

Many researchers have studied the fatigue behavior of Pb-based ferroelectrics in recent years.^9–13^ Many mechanisms and methods have been proposed to study the nature of electric fatigue: defect agglomeration,^14^ field screening resulting from surface damage,^15^ local phase decomposition^16^ and so on. Most of these mechanisms are based on these two steps: first, cyclic electric field induces a creation of imperfections or a redistribution of intrinsic imperfections; second, the subsequent imperfections affect the reversible polarization.^17^ Among all of the imperfections, point defects play an essential part because they affect the electric fatigue behavior significantly.^18,19^ The existence of point defects in materials also affects almost every physical property of the matrix. Almost all of the unsolved problems of ferroelectrics have some relations with defects, including aging,^20^ imprint^21,22^ and the unwanted increment of leakage current.^23^ It's of great significance to study the effect of point defects on ferroelectric materials. The most effective way to induce point defects is doping. Two kinds of point defects would be generated through doping: cation vacancies are generated *via* donor doping, and oxygen vacancies are generated through acceptor doping.^24^ Whether cation vacancies and oxygen vacancies play positive or negative roles in the fatigue behavior of bulk ferroelectrics is still unclear. Chen *et al.* reported that fatigue endurance improved more for donor-doped BaTiO_3_ ceramics than that of acceptor-doped materials.^25^ However, better fatigue endurance has been reported for acceptor-doped PbZrO_3_ ceramics than donor-doped PbZrO_3_ ceramics.^26^ Compared with the fatigue behavior of donor-doped ferroelectrics, it's more complicated in acceptor-doped ferroelectrics due to the high mobility of oxygen vacancies^27–30^ and the defect dipoles formed between defect ions and oxygen vacancies.^31^ The domain wall pinning due to the agglomeration of oxygen vacancies during the fatigue process hampers the motion of domain walls, leading to the detriment of fatigue endurance. While the defect dipoles formed in the bulk should reorient under strong bipolar electric field during the fatigue process, which results in more complicated fatigue behavior. Apart from the enhancement of fatigue endurance of acceptor-doped ferroelectrics mentioned above, some authors have found abnormal self-rejuvenation behavior of the remnant polarization (*P*_r_) during the fatigue process for poled and fully aged Pb-based ferroelectric ceramics under cyclic bipolar electric field due to the de-pinning or de-aging effect of oxygen vacancies.^32,33^ To our knowledge, this abnormal self-rejuvenation has not been found in un-poled and un-aged Pb-based ferroelectrics with perovskite structure, which has actually been found in this work. If well understood, the mechanism behind this phenomenon is bound to make great contribution to the materials science.

In this work, we focus on fatigue endurance enhancement of PLN–PT ceramics and study the effect of defect dipoles on electric fatigue behavior of acceptor-doped PLN–PT ceramics. 0.51PLN–0.49PT ceramic in the MPB region close to the rhombohedral side was chosen as the matrix in this work due to the excellent electric properties of this composition. It was found in the current work that for the composition 0.51PLN–0.49PT, the *P*_r_ decreases to half of the value measured at the beginning of the fatigue process before the number of cycles (*n*) increases up to 1.6 × 10^4^. This fatigue endurance is very weak compared with PMNT ceramics, the *P*_r_ of which keeps nearly stationary even up to 10^4^ cycles.^10^ Hence it is very necessary to enhance the fatigue endurance of 0.51PLN–0.49PT ceramics to make them more reliable. In this work, SnO_2_ was chosen as the dopant to obtain acceptor-doped 0.51PLN–0.49PT ceramics because there were many good works about Sn-doping in ferroelectrics like BaTiO_3_,^34^ Pb(Ti_0.65_Zr_0.35_)O_3_ (ref. 35) and BaNbO_3_.^36^ Sn was found to exist in oxides as both Sn^2+^ and Sn^4+^.^37–39^ We substituted Sn^4+^ ions for B-site Ti^4+^ ions. The valence change of Sn^4+^ thus led to the formation of oxygen vacancies to balance the charge misfit. To avoid the domain wall pinning of oxygen vacancies caused by aging effect, fatigue behavior was measured right after the annealing treatment. Ferroelectric, fatigue and dielectric characteristics of the samples with different Sn doping levels were studied, together with the chemical valence state of Sn and oxygen vacancies.

## 2. Experimental procedure

Sn ions modified PLN–PT ceramics with the formula of 0.51Pb(Lu_1/2_Nb_1/2_)O_3_–0.49PbTi_1−*x*_Sn_*x*_O_3_ (*x* = 0–0.2) were fabricated by the traditional solid-state reaction method using high purity (99.9%) PbO, Lu_2_O_3_, Nb_2_O_5_, TiO_2_, SnO_2_ as raw materials. Excess of 2 mol% of the total amount of PbO which was weighed according to the stoichiometry was added to compensate the evaporation of PbO during sintering. At first, all the raw materials were blended and ball-milled in ethanol with 2 mol% excess of PbO for 24 hours. Then the adequately milled powder was pressed into discs at 15 MPa and calcined at 850 °C in air for 3 hours. After calcination, the discs were remilled for 2 hours with 2 wt% polyvinyl alcohol (PVA) as a binder. Again, the powder was pressed into small pellets at 13 MPa, and the discs were heated up to 500 °C for 2 hours to eliminate PVA. At last, the discs were sintered at 930 °C to 970 °C for 3 hours in an Al_2_O_3_ crucible filled with PbZrO_3_ which prevents the excessive evaporation of PbO.

The crystalline phase of sintered specimens was analyzed by X-ray diffractometer (XRD) (Miniflex 600, Rikagu, Japan) from 5° to 80° (2*θ*) with a speed of 2 deg min^−1^ and a step of 0.02 (2*θ*) at room temperature. Theoretical density of the ceramics was calculated through the XRD profile using the software Jade 6.0. The observations of the microstructure of the fractured surfaces of the specimens were carried out by a field emission scanning electron microscope (SEM, Hitachi SU-8010, Japan). The sintered disc ceramics were cut into rectangle with the area about 8 mm^2^ to avoid edge effects, and polished to about 300 μm in thickness with 800 grit SiC papers, and then annealed at 500 °C for 3 hours to eliminate the aging effect. Right after the annealing, the samples were pasted with silver paste and then electrical measurements were made immediately. Ferroelectric characterizations and fatigue behavior were measured by the aixACCT TF Analyzer 2000 standard ferroelectric test system. Before the fatigue treatment, the polarization–electric field (*P*–*E*) hysteresis loops were measured under an AC field of 5 Hz with the amplitude of 60 kV cm^−1^. It should be noted that the amplitude of the applied field was enhanced slowly in order not to break the sample. Then the samples were fatigued immediately. For fatigue behavior, the samples were subjected to a bipolar AC field with the amplitude of about 2 times of the coercive field (*E*_C_) (determined before the fatigue treatment) and frequency of 100 Hz up to 3 × 10^6^ cycles. Dielectric properties measurements were performed by Alpha-A high resolution impedance analyzer (Novocontrol GmbH). Oxygen vacancies and the oxidation states of Sn ions were investigated by X-ray photoelectron spectroscopy (XPS, ESCALAB 250Xi). The samples for XPS investigation were all polished to eliminate the influence of the surface state.

## 3. Results and discussion

### 3.1 Structure analysis

**Fig. 1:**
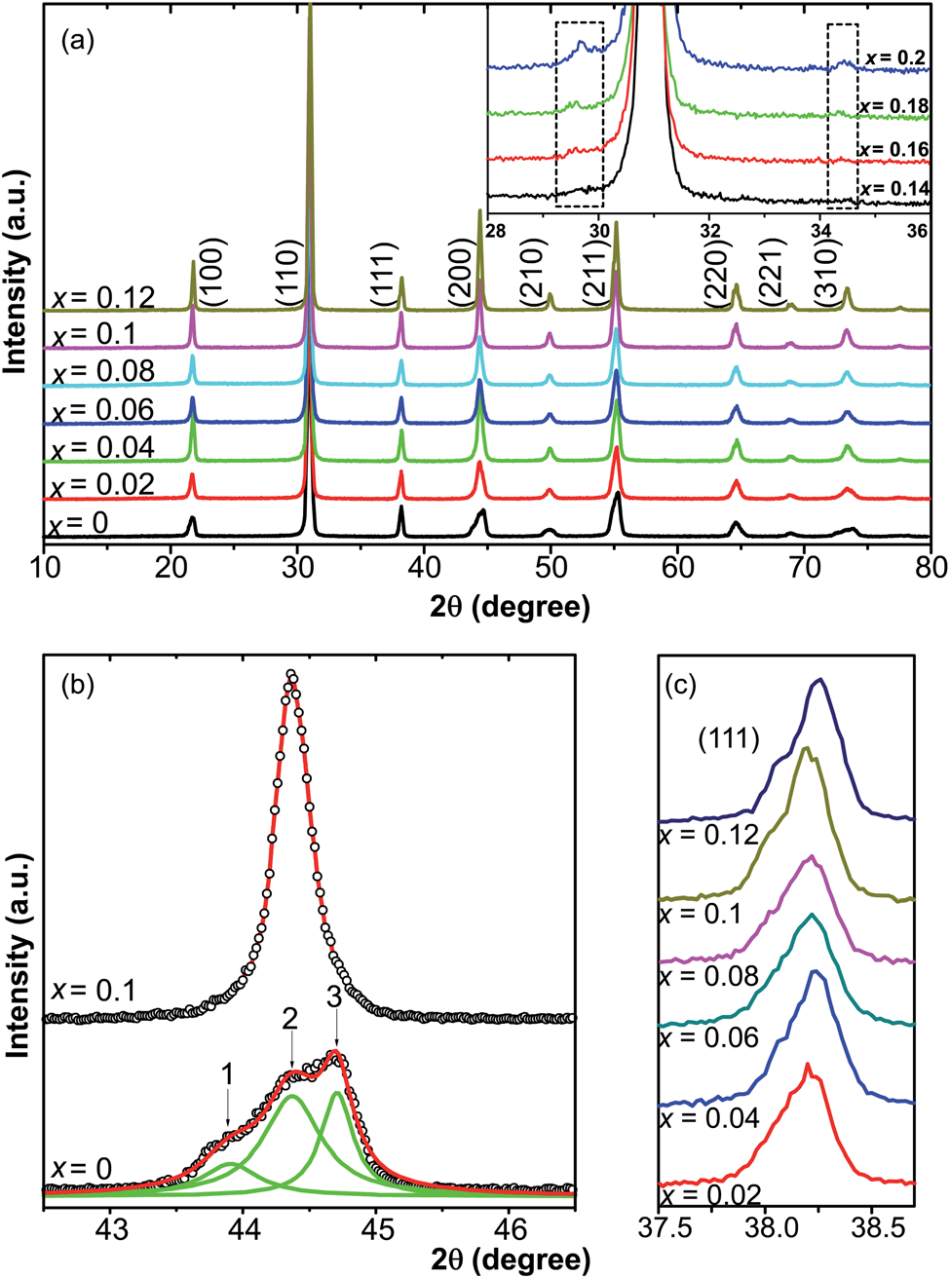
(a) XRD patterns of 0.51Pb(Lu_1/2_Nb_1/2_)O_3_–0.49PbTi_1−*x*_Sn_*x*_O_3_ ceramics with *x* = 0–0.12. The inset shows the details between 28 and 36 degree, indicating a secondary phase. (b) (200)_C_ profile of *x* = 0 and *x* = 0.1, showing that the dopant of Sn changed the symmetry from an MPB region to a pure rhombohedral phase, erasing the tetragonal phase. (c) The profile of (111) peak with *x* = 0.02–0.12.

The XRD patterns of 0.51Pb(Lu_1/2_Nb_1/2_)O_3_–0.49PbTi_1−*x*_Sn_*x*_O_3_ ceramics with compositions of *x* = 0–0.2 at room temperature are presented in Fig. 1(a), showing perovskite structures with *x* = 0–0.12 and small amount of secondary phase compositions with *x* > 0.14 (the XRD profiles of which were enclosed by two dashed squares in the inset of Fig. 1(a)), which was found to be Sn_2_Nb_2_O_7_ and cannot be eliminated easily by changing synthesis conditions. The pyrochlore phase was caused by the formation of Sn^2+^. The appearance of Sn^2+^ made it easy to form the Sn_2_Nb_2_O_7_ pyrochlore phase with Nb^5+^ ions. The valence state of Sn will be discussed later in the current article. In order to study the effect of Sn ions on the structure of PLN–PT ceramics, attention was paid to the XRD profiles of (200)_C_ around 2*θ* = 45°. Shown in Fig. 1(b) were the profiles of (200) peaks of the composition of *x* = 0 and *x* = 0.1. For the pure PLN–PT (*x* = 0) ceramics, the (200) peak around 2*θ* = 45° showed an asymmetrical peak, composed of three peaks with Gauss distribution. The ratio of the intensities of peak 1 and peak 3 is about 1 : 2. It is known that the profile of R(200) peak of rhombohedral (R) structure exhibits only a single sharp symmetrical peak because for rhombohedral phase, the relation among the lattice parameters is *a* = *b* = *c*, making the patterns of (200) and (002) peaks indistinguishable. While for the tetragonal (T) symmetry, the (200) peak ought to split into two peaks, corresponding to T(200)/(020) and T(002) with the ratio of the intensities of the two peaks about 2 : 1 because of *a* = *b* ≠ *c*.^40,41^ Obviously, peak 2 corresponds to the rhombohedral phase. Peak 1 and 3 correspond to the tetragonal phase. The (200)_C_ (subscript C represents cubic) peak of all the doped compositions is sharper and more symmetrical than that of the virgin composition. Shown in Fig. 1(c) were the profile of (111) peak from *x* = 0.02 to *x* = 0.12. It could be seen that all the peaks showed asymmetric profile, which is a characteristic of rhombohedral structure.^42^ So the dopant of Sn changed the symmetry from an MPB region to a rhombohedral phase, eliminating the tetragonal phase.

**Fig. 2:**
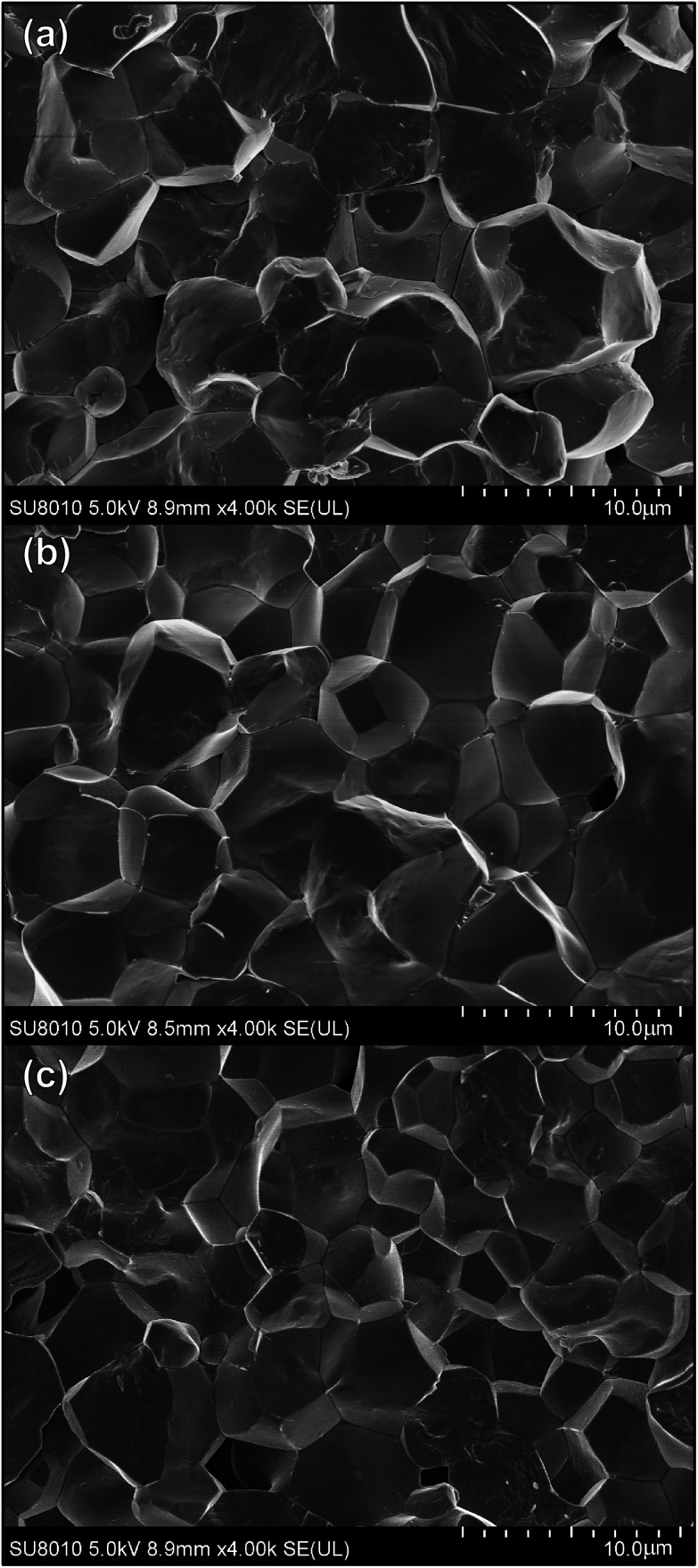
SEM micrographs of fractured surfaces of 0.51Pb(Lu_1/2_Nb_1/2_)O_3_–0.49PbTi_1−*x*_Sn_*x*_O_3_ ceramics: (a) *x* = 0; (b) *x* = 0.04; (c) *x* = 0.08.

The SEM micrographs of the selected ceramics (*x* = 0, 0.04, 0.08) are shown in Fig. 2, showing highly dense structure with clear grain boundaries, indicating intergranular structure. Theoretical density of the refined XRD data was calculated and the relative density of each sample without secondary phase is more than 90% of the theoretical density, namely, the low level of porosity. The average size of grains for the virgin specimens is about 7–9 μm. The grains shrink slightly with the increase of *x* with about 4–5 μm for *x* = 0.08. So the substitution of Sn for Ti ions has significant influence on the morphology of PLN–PT ceramics.

### 3.2 Ferroelectric properties

**Fig. 3:**
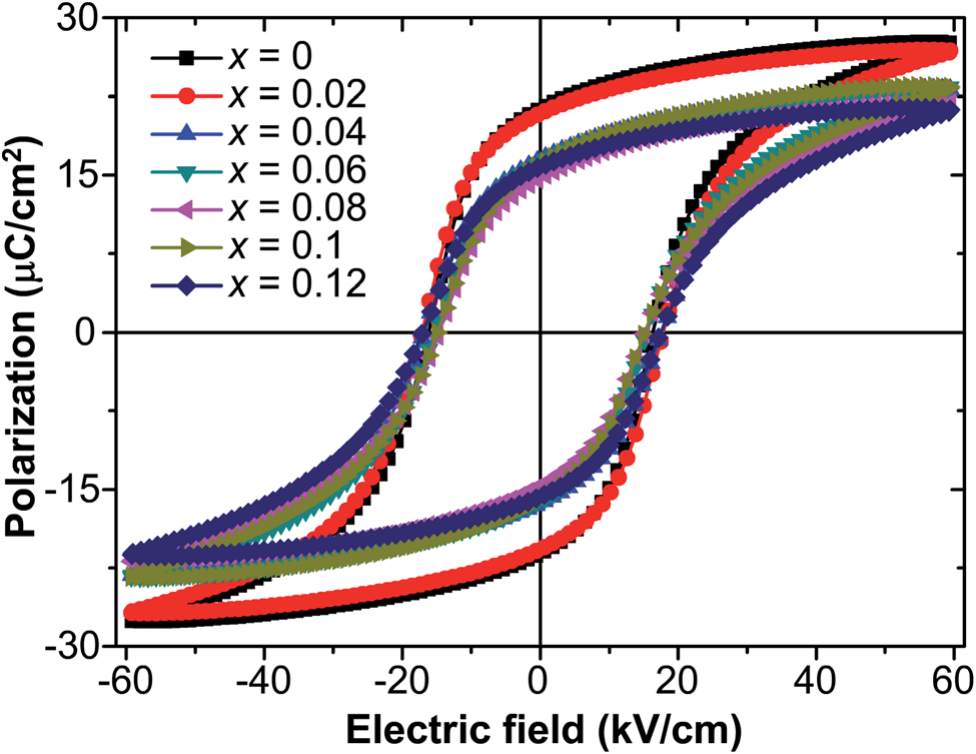
*P*–*E* hysteresis loops of 0.51Pb(Lu_1/2_Nb_1/2_)O_3_–0.49PbTi_1−*x*_Sn_*x*_O_3_ ceramics.

Fig. 3 presents the *P*–*E* hysteresis loops of the selected Sn-doped and un-doped PLN–PT ceramics. Well-saturated loops were found for every sample, indicating good ferroelectricity. The squareness (*R*_sq_) of the loops quantifies the ferroelectricity from one aspect, and could be calculated *via* the following equation:^43^1
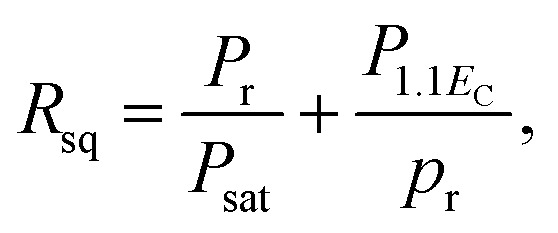
where *P*_r_ is the remnant polarization, defined as |*p*^+^_r_ − *p*^−^_r_|/2 in the current work. *p*^+^_r_ and *p*^−^_r_ are the positive and negative value of *P*_r_, respectively. *P*_sat_ is the saturation polarization. *P*_1.1*E*_C__ is the polarization at the electric field 1.1 times of *E*_C_, defined as |*E*^+^_C_ − *E*^−^_C_|/2 in the current work. *E*^+^_C_ and *E*^−^_C_ are the positive and negative values of *E*_C_, respectively. The values of *P*_r_, *P*_sat_, *P*_1.1*E*_C__, *E*_C_ and *R*_sq_ for every selected sample were listed in Table 1. For a perfect loop, *R*_sq_ = 2. It can be seen that *R*_sq_ for the studied samples in this work are all larger than 1.91 and show little fluctuation with the variance of *x*, showing good squareness of the loops.

**Table 1:** Ferroelectric properties of 0.51Pb(Lu_1/2_Nb_1/2_)O_3_–0.49PbTi_1−*x*_Sn_*x*_O_3_ ceramics

*x*	*P*_r_ (μC cm^−2^)	*P*_sat_ (μC cm^−2^)	*P*_1.1*E*_C__ (μC cm^−2^)	*E*_C_ (kV cm^−1^)	*R*_sq_
0	21.2	27.6	25.0	16.2	1.95
0.02	20.7	26.8	24.6	17.5	1.96
0.04	16.3	23.2	20.4	17.3	1.95
0.06	16.1	24.0	20.1	15.4	1.92
0.08	14.6	22.8	18.6	14.9	1.91
0.1	15.9	23.8	20.2	15.0	1.94
0.12	15.7	21.7	19.1	17.3	1.94

**Fig. 4:**
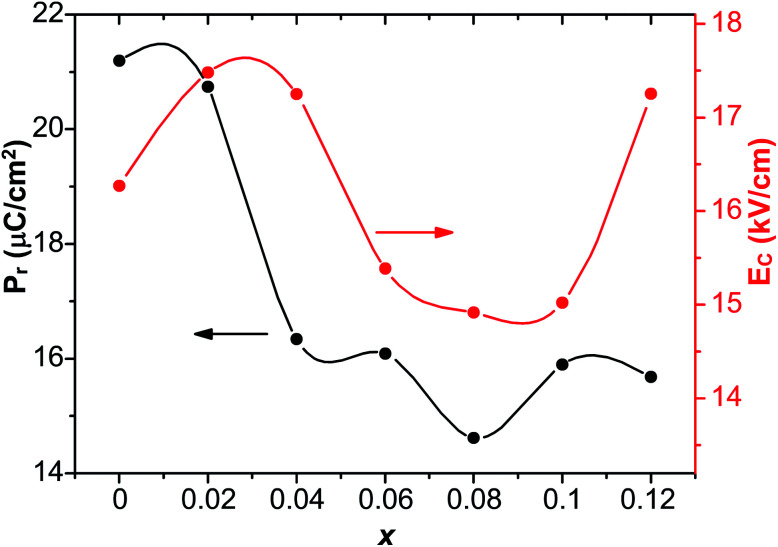
The values of *P*_r_ and *E*_C_ of 0.51Pb(Lu_1/2_Nb_1/2_)O_3_–0.49PbTi_1−*x*_Sn_*x*_O_3_ ceramics with respect to the content of Sn.

To investigate the relation between the content of Sn and ferroelectric properties, the variation of *P*_r_ and coercive field *E*_C_ in terms of the content of Sn is depicted in Fig. 4. *P*_r_ drops from *x* = 0 to *x* = 0.04, then changes little with *x* increasing. *E*_C_ drops to the minimum at *x* = 0.08, then magnifies with more the content of Sn. The decrement of *P*_r_ and *E*_C_ at low-Sn-ion region is attributed to the decrease of the content of PbTiO_3_ (PT). The magnifying of *E*_C_ at *x* > 0.08 mainly results from the effect of defect dipoles formed as 
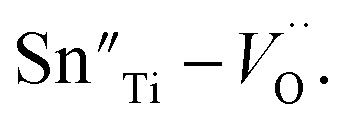
 It is found that when calcined at high temperature, the Sn^4+^ of SnO_2_ partly reduces to Sn^2+^ to form a mix state of the valence of +4 and +2.^39^ The multivalent behavior of Sn has already been investigated previously.^44,45^ The evidence of the change of valence state of Sn ions in this work will be presented below. The change of valence state from +4 to +2 thus leads to the formation of oxygen vacancies to balance the charge misfit. As a result, defect dipoles 
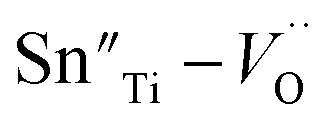
 were formed. At *x* < 0.1, oxygen vacancies were not dense enough to have conspicuous effect on the materials, on which the decrease of the content of PT have more pronounced influence. At *x* > 0.08, enough oxygen vacancies were generated to form defect dipoles, leading the domain stability to be strengthened by the effect of defect dipoles. This effect stemmed from a symmetry-conforming property of point defects.^31^ The irreversible defect dipoles make the switching of spontaneous polarization much harder, resulting in an increment of *E*_C_ at *x* > 0.08.

**Fig. 5:**
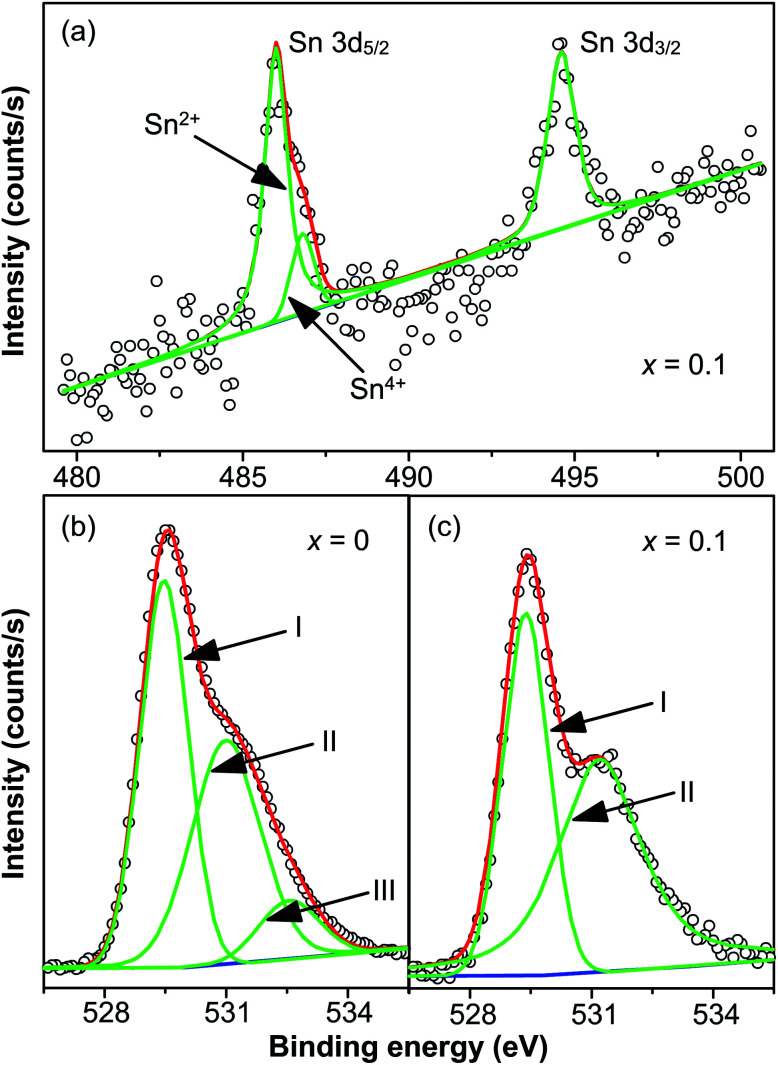
(a) XPS spectrum of Sn 3d core level of *x* = 0.1 at room temperature, and XPS spectrum of O 1s core level of (b) *x* = 0 and (c) *x* = 0.1 at room temperature. Circles were the measured spectra, red curves were the fitted curves and green curves were the individual Gaussian peaks and blue lines are the Tougaard-type background.

The chemical valence state of Sn and oxygen vacancies was examined X-ray photoelectron spectroscopy. Fig. 5(a) shows the XPS spectrum of Sn 3d core level of *x* = 0.1 at room temperature, showing two peaks in the binding energy range between 480 and 500 eV. The two peaks centered at 486.02 and 494.6 eV are for Sn 3d_5/2_ and Sn 3d_3/2_ respectively. Interestingly, two peaks centered at 486.02 and 486.84 eV are displayed after the fitting of the Sn 3d_5/2_ peak. It is reported that the two peaks of Sn 3d_5/2_ peak correspond to two valence states: Sn^2+^ (486.2 eV) and Sn^4+^ (487.1 eV).^39^ Hence it is believed that both Sn^2+^ (486.02 eV) and Sn^4+^ (487.1 eV) exist in doped samples. The intensity of Sn^2+^ peak was much larger than the peak of Sn^4+^, indicating that most Sn^4+^ ions were reduced to Sn^2+^ during the high-temperature calcination. This also supports the result discussed above that a pyrochlore phase with Sn_2_Nb_2_O_7_, in which the valence of Sn ions is +2, is formed as a secondary phase at high-level dopant.

The reduction of the valence state of Sn ions leads to the formation of oxygen vacancies to compensate the misfit of charge, which is demonstrated by the XPS of O 1s core level of *x* = 0 and *x* = 0.1 shown in Fig. 5(b) and (c). It should be noted that in Fig. 5, circles are the measured spectra, red curves are the fitted curves, green curves are the fitted Gaussian peaks and blue lines are the Tougaard-type background. The spectrum of *x* = 0 is well fitted with three Gaussian peaks with a Tougaard-type background, plotted as blue curves in the figures. The peaks are located at 529.46 eV (O_I_), 531 eV (O_II_) and 532.54 eV (O_III_). The spectrum of *x* = 0.1 is well fitted with two Gaussian peaks with a Tougaard-type background. The centers of the peaks are centered at 529.38 eV (O_I_) and 531.24 eV (O_II_). Peak O_I_ is associated with oxygen ions incorporated in the lattice sites in materials with perovskite structure. Peak O_II_ is attributed to the formation of oxygen vacancies. Peak O_III_, which was not found in the spectrum of *x* = 0.1, resulted from the chemisorbed oxygen or dissociated oxygen.^46^ Similar cases were also reported in several works.^28,47–49^ The ratio of the area of O_II_ and O_I_ (denoted as *R*_a_) was also calculated to determine the density of oxygen vacancies. For *x* = 0, *R*_a_ = 0.85, while for *x* = 0.1, *R*_a_ = 1.28, suggesting that oxygen vacancies of *x* = 0.1 are denser than that of *x* = 0. Hence the reduction of valence state of Sn^4+^ ions leads to the formation of oxygen vacancies, and defect dipoles are formed.

### 3.3 Fatigue behavior

**Fig. 6:**
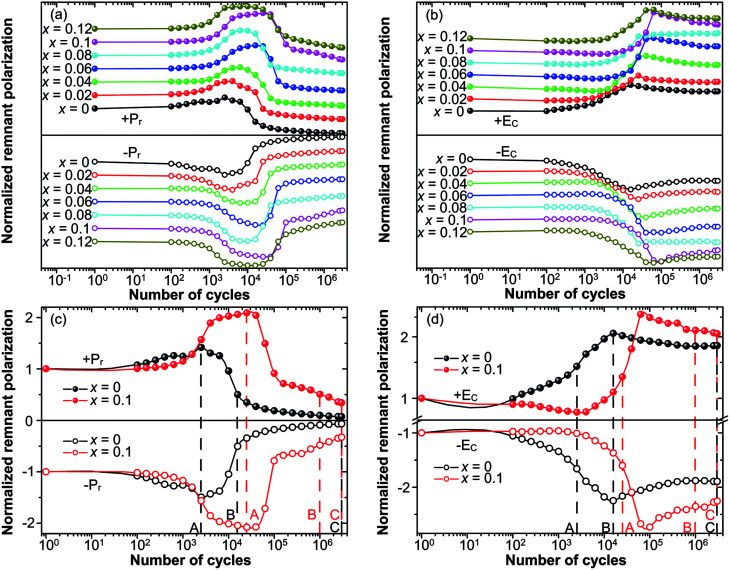
Plots of (a) normalized ±*P*_r_ and (b) normalized ±*E*_C_ as a function of the number of cycles for 0.51Pb(Lu_1/2_Nb_1/2_)O_3_–0.49PbTi_1−*x*_Sn_*x*_O_3_ ceramics under an applied bipolar field of 2*E*_C_ for each composition at 100 Hz. Normalized *P*_r_ and normalized *E*_C_ of the composition *x* = 0 and *x* = 0.1 were extracted and shown in (c) and (d) respectively.

The fatigue behavior was studied as shown in Fig. 6. Fig. 6(a and b) show the variation of *P*_r_ and *E*_C_ of all of the compositions measured at different *n*. For comparison, *P*_r_ and *E*_C_ were normalized to the corresponding values measured at the first circle. It can be easily seen that the curves of *P*_r_ and *E*_C_ of the studied samples show the similar trends. For precise study, the extracted curves of *x* = 0 and *x* = 0.1 compositions are shown in Fig. 6(c and d). *P*_r_ of both *x* = 0 and *x* = 0.1 shows an abnormal self-rejuvenation in the middle of the fatigue process, different from normal electric fatigue behavior of ferroelectrics, which doesn't show increment before decrement.^12,50–53^*E*_C_ of *x* = 0 first increased then decreased, with peak value located near point A. For *x* = 0.1, *E*_C_ experienced a decrement before increasing to the peak value, after which decreased. Generally, an increase of *E*_C_ was observed after the fatigue treatment for bulk ferroelectrics.^54^ The abnormal behavior of *E*_C_ for *x* = 0.1 and *P*_r_ shall be discussed later.

**Fig. 7:**
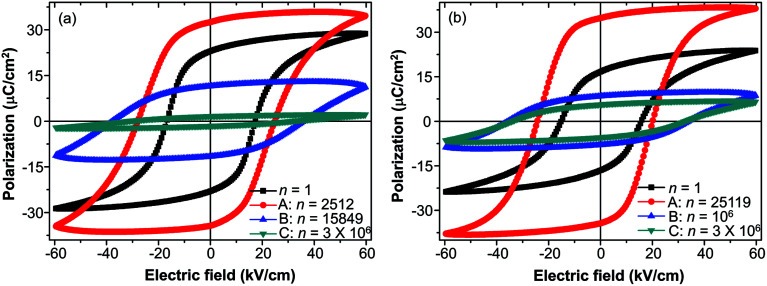
*P*–*E* hysteresis loops of (a) *x* = 0 and (b) *x* = 0.1 at different points: A, at which *P*_r_ increases to its maximum; B, at which *P*_r_ decreases to below 50% of the value of *n* = 1; C, at the end of the fatigue process.

The study of fatigue behavior of Sn-doped PLN–PT ceramics indicate that the fatigue endurance is enhanced greatly with Sn doped. In the following, we give some elucidation. Special attention was focused on: point A, at which *P*_r_ reaches maximum; point B, at which *P*_r_ decreases to half of the value of the first circle; and point C, at the end of the fatigue process, 3 × 10^6^ cycles in the current work. To evaluate the fatigue endurance, the variation of the values of *P*_r_ and *E*_C_ at point B is listed in Table 2, indicating enhanced fatigue endurance of doped samples, because the value of *P*_r_ of all of the doped samples drops to half at more cycles than that of undoped samples. Compared with *x* = 0 (*n* = 15 849), the cycles are *n* = 10^6^ for *x* = 0.1, which is elevated by two orders of magnitude. The variation of the value of *P*_r_ and *E*_C_ of *x* = 0 and *x* = 0.1 compositions at point A, B, C are listed in Table 3. At point A, the value of *P*_r_ increases 45.6% for *x* = 0, while there is a 108.38% augment of *P*_r_ for *x* = 0.1. To have a direct understanding, the *P*–*E* hysteresis loops of *x* = 0 and *x* = 0.1 at three points and first cycle (*n* = 1) are presented in Fig. 7. At *n* = 1 for both compositions, the loops are not pinched or displaced, characteristic of the un-poled and un-aged case. For *x* = 0, the squareness increases from 1.96 to 1.97 from *n* = 1 to *n* = 2512, and the squareness shows an increment from 1.95 to 1.995 from *n* = 1 to *n* = 25 119 for *x* = 0.1. The improvement of the squareness of *x* = 0.1 is greater than that of *x* = 0, apart from the more improvement of *P*_r_ value. It could also be seen that after 3 × 10^6^-cycle fatigue treatment, the *P*–*E* hysteresis loop of *x* = 0 almost withers to a straight line clinging to the *x*-axis, while the loops of *x* = 0.1 still retained a moderate shape. This also helped elucidate the enhancement of fatigue endurance of the material *via* Sn doping.

**Table 2:** Fatigue behavior of 0.51Pb(Lu_1/2_Nb_1/2_)O_3_–0.49PbTi_1−*x*_Sn_*x*_O_3_ ceramics at the point at which *P*_r_ began to decrease to half of *P*_r_ measured at *n* = 1

*x*	*n* (cycles)	% change of *E*_C_
0	15 849	116.2
0.02	24 821	95.6
0.04	35 558	127.5
0.06	68 013	150.2
0.08	100 096	116.8
0.1	10^6^	125.1
0.12	371 735	88.8

**Table 3:** Fatigue behavior of ferroelectric properties for *x* = 0 and *x* = 0.1 at different number of cycles^a^

*x*	*n* (cycles)	*P*_r_ (μC cm^−2^)	*E*_C_ (kV cm^−1^)	% change of *P*_r_	% change of *E*_C_	Note
0	2512	33.4	26.3	45.6	54.4	A
	15 849	11.6	36.8	−49.7	116.2	B
	3 × 10^6^	1.6	31.6	−93.1	85.2	C
0.1	25 119	34.6	22.2	108.4	43.5	A
	10^6^	8.2	34.8	−50.6	125.1	B
	3 × 10^6^	5.5	33.4	−66.6	116.2	C

a A, *P*_r_ increases to its maximum; B, *P*_r_ decreases to below 50% of the value of *n* = 1; C, the end of the fatigue process.

As above mentioned, the abnormal behavior of *P*_r_ and *E*_C_ is attributed to the effect of the reorientation of defect dipoles. The abnormal increment has also been reported for acceptor doped PZT-based ceramics.^32,33^ But the PZT samples studied before fatigue treatment were poled and fully aged. Such abnormal behavior, to our knowledge, has not been reported for un-poled and un-aged acceptor-doped ferroelectric materials with perovskite structure. It is known that oxygen vacancies have high mobility. Under strong electric field, charged defect dipoles can migrate even at a relatively low temperature.^55^ Defect dipoles thus reorient and become more reversible under strong bipolar electric field. The reorientation and enhanced reversibility of defect dipoles under the bipolar field make the switching of spontaneous polarization much easier.^32^ The release of spontaneous polarization from defect dipoles thus leads to the increase of *P*_r_ (for both *x* = 0 and *x* = 0.1) and decrease of *E*_C_ (for *x* = 0.1) at the first half part of the fatigue process. The fact mentioned above that *P*_r_ of *x* = 0.1 increases much more than that of *x* = 0 at point A suggests that more defect dipoles were generated and reoriented in the sample of *x* = 0.1, supporting the conclusion we drew above that the dopant of Sn ions leads to the formation of oxygen vacancies. The decrease of *P*_r_ and *E*_C_ after point A was the result of the field-shielding effect, resulting from the formation of layers with weak ferroelectric and dielectric properties under large number of cyclic electric field. And it is clear that in the fatigue process the reorientation of defect dipoles dominated before point A and field-shielding effect dominated after point A.

**Fig. 8:**
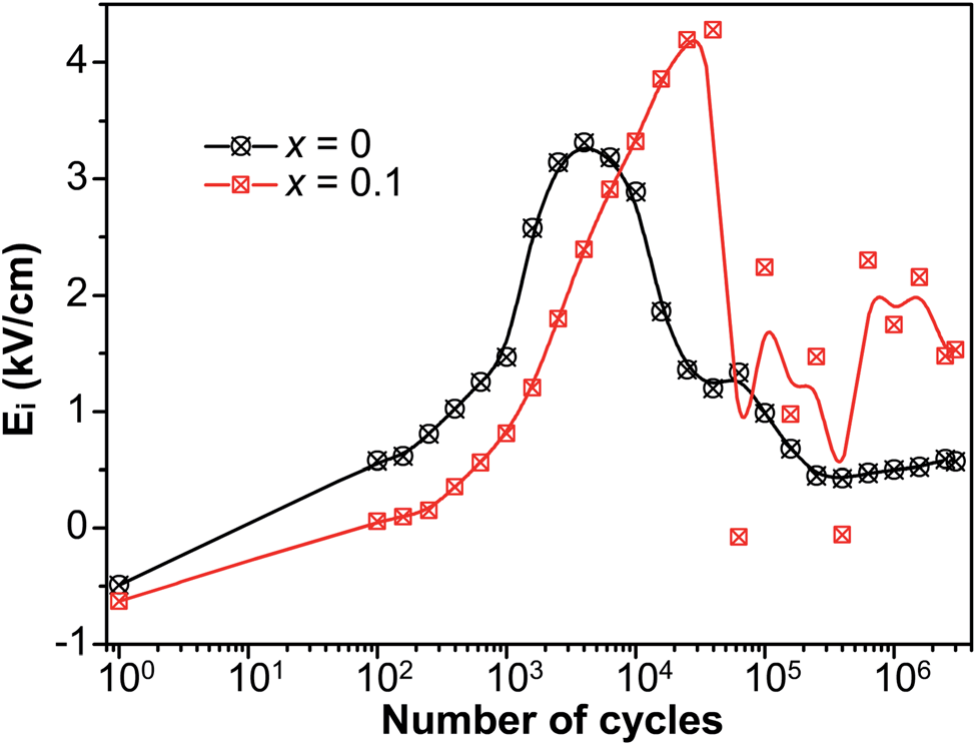
The internal bias (*E*_i_) of *P*–*E* hysteresis loops *versus* the number of cycles (*n*) during the fatigue process.

It is well known that there exists a correlation between the magnitude of internal bias field and the alignment of defect dipoles.^56^ To further manifest the reorientation of defect dipoles, the internal bias with respect to *n* during the fatigue process is plotted in Fig. 8. The internal bias field in the current work is defined as *E*_i_ = (*E*_−_ − *E*_+_)/2, where *E*_+_ and *E*_−_ are the magnitude of the intersections of the *P*–*E* hysteresis loops and the electric field axis. *E*_i_ of both *x* = 0 and *x* = 0.1 increased to maximum near point A. The maximum of *E*_i_ for *x* = 0.1 appear at more cycles than that of *x* = 0, and the peak value of *E*_i_ of *x* = 0.1 is also larger than that of *x* = 0. It suggests that much more defect dipoles are formed and reorient in the sample of *x* = 0.1. In addition, a rather drastic fluctuation was detected of *x* = 0.1 after reaching the maximum. It is due to the competition between the reorientation of defect dipoles and the field-shielding effect, and it is this competition that leads to the enhancement of fatigue endurance of fatigue endurance.

### 3.4 Dielectric properties

**Fig. 9:**
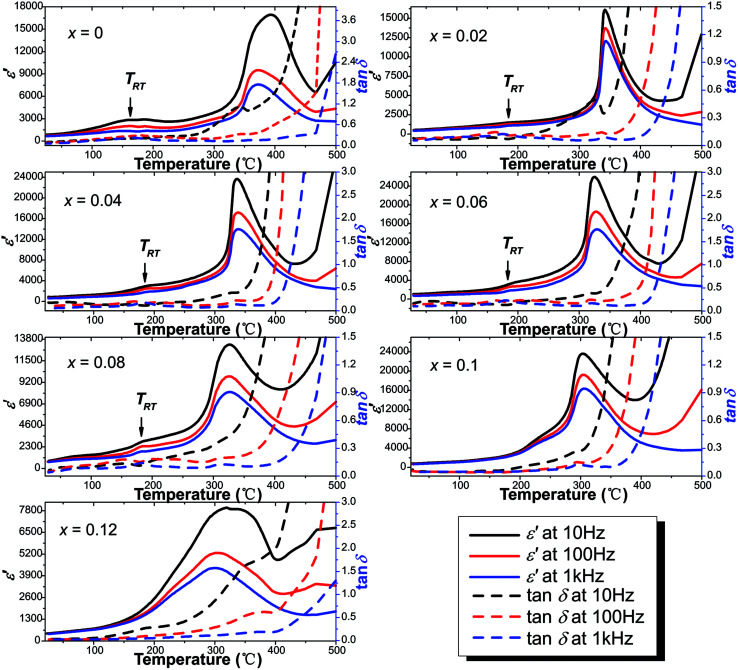
Temperature dependence of the real part of dielectric constant and dielectric loss.

**Fig. 10:**
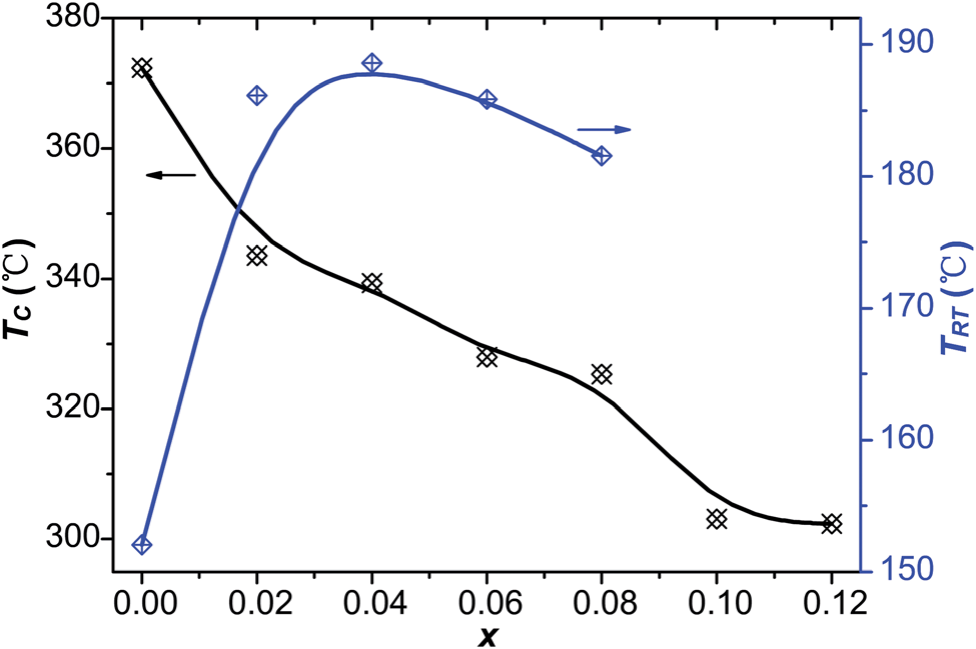
The variation of *T*_RT_ and *T*_C_ in terms of *x*.

Fig. 9 shows the temperature dependence of the real part of the relative dielectric constant (*ε*′) and the dielectric loss (tan *δ*) of the virgin and Sn-doped PLN–PT ceramics at the frequency of 10 Hz, 100 Hz and 1k Hz, respectively. Two peaks were detected in samples with *x* ≤ 0.08. The sharper one corresponds to Curie temperature *T*_C_, and the lesser one below *T*_C_ is known as rhombohedral–tetragonal transition temperature, denoted as *T*_RT_. For samples with *x* ≥ 0.1, *T*_RT_ disappears. The values of *ε*′, tan *δ*, *T*_RT_ and *T*_C_ of the studied compositions were listed in Table 4. The variation of *T*_RT_ and *T*_C_ with respect to the content of Sn is shown in Fig. 10. It can be seen that *T*_C_ decreases from 372 °C to 302 °C as *x* increases. *T*_RT_ first increases, reaching maximum at *x* = 0.04, then decreases as *x* increases to 0.08, after which *T*_RT_ disappears. It is found that the trend of *T*_C_ and *T*_RT_ of this work fits well to that of PLN–PT crystals that Liu *et al.* reported,^4^ in which *T*_C_ of PLN–PT also decrease as the content of PT decreases, and the *T*_RT_ also shows a small peak around the composition 0.55PLN–0.45PT.

**Table 4:** Dielectric properties of 0.51Pb(Lu_1/2_Nb_1/2_)O_3_–0.49PbTi_1−*x*_Sn_*x*_O_3_ ceramics

*x*	*ε*′ at RT	tan *δ* at RT	*T*_RT_ (°C)	*T*_C_ (°C)
0	869.2	0.09	152.0	372.3
0.02	524.4	0.07	186.2	343.5
0.04	844.8	0.17	188.6	339.4
0.06	1060	0.16	186.0	327.9
0.08	795.4	0.06	181.6	325.3
0.1	759.3	0.07	—	303.1
0.12	450.1	0.03	—	302.4

## 4. Conclusions

In summary, the structure, electric fatigue behavior and related electric properties of 0.51Pb(Lu_1/2_Nb_1/2_)O_3_–0.49PbTi_1−*x*_Sn_*x*_O_3_ ceramics were studied. The valence state of Sn was examined by XPS to change from +4 to +2 during high-temperature calcination so that the oxygen vacancies were created to balance the charge misfit. At high level of Sn content, defect dipoles caused by Sn ions and oxygen vacancies induce an increment of *E*_C_. The electric fatigue endurance is enhanced after the doping of Sn. The best fatigue endurance is achieved at composition *x* = 0.1, *n* of which was elevated by two orders of magnitude. An abnormal strong self-rejuvenation of the *P*_r_ was also detected for un-poled and un-aged samples. The enhancement of fatigue endurance and abnormal self-rejuvenation result from the reorientation of defect dipoles 
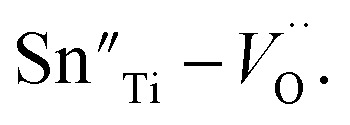
 The temperature dependence of the relative dielectric constant of different compositions shows that the *T*_C_ decreased as the content of Sn increased and the *T*_RT_ decreased after an increment at *x* < 0.04. In this work, we gave evidence that defect dipoles not only play a positive role in the electric fatigue behavior of un-poled and un-aged acceptor-doped ferroelectric materials with perovskite structure, but also induce an abnormal self-rejuvenation behavior during the fatigue process.

## Conflicts of interest

There are no conflicts to declare.

## Supplementary Material
